# Glucose-Free Solutions Mediated Inhibition of Oxidative Stress and Oxidative Stress-Related Damages in Peritoneal Dialysis: A Promising Solution

**DOI:** 10.3390/life14091173

**Published:** 2024-09-18

**Authors:** Anna Basso, Martina Cacciapuoti, Lucia Federica Stefanelli, Federico Nalesso, Lorenzo A. Calò

**Affiliations:** Nephrology, Dialysis and Transplantation Unit, Department of Medicine, University of Padova, 35128 Padova, Italy; anna.basso@aopd.veneto.it (A.B.); martina.cacciapuoti@phd.unipd.it (M.C.); luciafederica.stefanelli@unipd.it (L.F.S.); federico.nalesso@unipd.it (F.N.)

**Keywords:** oxidative stress, chronic kidney disease, peritoneal dialysis, icodextrin, glucose-free solutions, osmo-metabolic agents, XyloCore, nephrology

## Abstract

Oxidative stress (OxSt) and inflammation are common in end-stage renal disease and dialysis patients; they are known risk factors for cardiovascular disease and mortality. In peritoneal dialysis (PD), OxSt and inflammation are even further increased compared to the already increased oxidative stress of their pre-dialysis phase. This is due to the high glucose-based solutions currently used, whose continuous contact with the peritoneal membrane can induce significant long-term morphological and functional changes (mesothelial to mesenchymal transition, thickening, neo-angiogenesis and fibrosis) of the peritoneal membrane. Oxidative stress plays a very important role in these processes, which may compromise the peritoneal dialysis procedure. There is, therefore, the need for more biocompatible dialysis fluids with polymers other than glucose to prevent and treat OxSt and inflammation. The most known and used of such glucose-free and more biocompatible peritoneal dialysis solutions is icodextrin, which has shown a protective effect from oxidative stress. This has supported the consideration of the use of glucose-free-based peritoneal dialysis fluids in order to reduce oxidative stress and improve peritoneal membrane survival. Studies investigating peritoneal dialysis with the use of osmo-metabolic agents (L-carnitine, xylitol and their combination) in peritoneal fluids replacing glucose-based fluids are, in fact, ongoing. They represent a promising strategy to reduce OxSt, preserve the peritoneal membrane’s integrity and improve patients’ outcome.

## 1. Introduction

Cardiovascular disease is known to be the leading cause of morbidity and mortality in patients with end-stage renal disease (ESRD). Traditional risk factors such as hypertension, diabetes, dyslipidemia, obesity and metabolic syndrome add to nontraditional risk factors, predisposing patients to cardiovascular (CV) disease, vascular calcification-accelerated atherogenesis and anemia [1]. Increased oxidative stress (OxSt), endothelial dysfunction and inflammation, further amplified by renal replacement procedures, may mediate most of the effects of these risk factors in ESRD patients [1,2]. Therefore, in ESRD patients, it becomes very important to be able to reduce these potentially modifiable risk factors.

OxSt is defined as the altered balance between oxidative molecules and reduced antioxidant defenses in favor of the former. This results in the alteration of intra-cellular redox homeostasis [3], tissue injury and systemic damage. Among the endogenous pro-oxidant factors, reactive oxygen species (ROS) are an important class of compounds produced during mitochondrial oxidative phosphorylation or from other pathways. A representative ROS is superoxide (O_2_^−^), which is produced by the nicotinamide adenine dinucleotide phosphate (NADPH) oxidase, uncoupled nitric oxide synthase, xanthine oxidase, cytochrome P450, lipoxygenase and cyclooxygenase. O_2_^−^ might immediately or catalytically dismutate to hydrogen peroxide (H_2_O_2_), another more stable ROS, which is able to cross membranes. H_2_O_2_ is catalytically decomposed to a hydroxyl radical (OH^−^) and together with O_2_^−^, acts as a signaling molecule. In addition, O_2_^−^ induces the production of other compounds such as radical nitric oxide (NO^−^) and peroxinitrites (ONOO^−^), which are toxic.

The altered redox homeostasis by the uncontrolled higher reactivity of ROS damages essential biomolecules such as lipids (injury of biological membranes) [4], proteins (fragmentation/aggregation) and DNA (altered gene expression). For example, in lipids, OH^•^ abstracts an H^+^, producing a free radical, which, in turn, binds to molecular oxygen (O_2_), triggering an amplification of redox reactions producing radical lipid peroxides, which affects cellular membranes producing malondialdehyde (MDA), which was found to exert an important role in the onset of atherogenesis [5].

All these abnormalities result in the onset and progression of endothelial dysfunction, atherosclerosis and inflammatory disease, acting as nontraditional risk factors in the development of renal and cardiovascular disease [1]. The multiple interconnections between OxSt, inflammation and endothelial dysfunction [1] result, in fact, in a vicious circle that exacerbates the underpinning processes between cardiovascular and kidney disease, finally resulting in the high morbidity and mortality of these patients [1].

## 2. Oxidative Stress in Peritoneal Dialysis (PD)

In chronic kidney disease (CKD) and dialysis patients, increased oxidative stress is caused by the activation of NADPH oxidase and the activation of the RhoA/Rho kinase (ROCK) pathway. Both of these are deeply involved in the OxSt-mediated cardiovascular risk/disease, while cardiovascular protection is provided by the inhibition of RhoA/ROCK signaling [1,6].

The role of oxidative stress and ROCK signaling and the positive effects of their reduction in chronic hemodialysis patients have been clearly shown. In some specific hemodialysis procedures, such as hemodiafiltration with the online reinfusion of ultrafiltrate [7], the use of vitamin E-coated dialyzers [8] have proven to be very effective in reducing OxSt.

Studies in PD patients are very few and have shown an increase in OxSt. PD is another renal replacement therapeutic option that is available for ESRD patients, representing an important alternative to hemodialysis, offering more flexibility, allowing patients to continue working, preserving patients’ residual renal function and having a lower cardiovascular impact [9,10]. PD is based on the infusion of a sterile solution through a permanent catheter into the abdomen and uses the peritoneal membrane as the exchange surface. The infused solution, which is hyperosmolar to plasma due to the addition of osmotic agents (most commonly glucose), is in close contact with the capillaries in the peritoneum, allowing for the removal of toxins, fluids and electrolytes in excess from the blood, before being removed itself. This may either occur at regular intervals throughout the day—continuous ambulatory peritoneal dialysis (CAPD)—or at night with the assistance of a machine—automated peritoneal dialysis (APD) or continuous cycling peritoneal dialysis (CCPD). However, continuous contact with the high glucose concentration present in the current PD fluids during PD can induce significant alterations of peritoneum in terms of morphology and functionality with OxSt playing a very important role [11,12,13].

OxSt in PD patients is likely induced by the composition of conventional PD solutions, which have a high glucose concentration, increased osmolarity and an acidic pH. These characteristics make these solutions clearly non physiological.

OxSt, in fact, follows the start of peritoneal dialysis, as demonstrated by increased advanced glycation end products (AGEs) and other pro-oxidant glucose byproducts [11,12,13]. AGEs and other pro-oxidant glucose degradation products (GDPs) are formed in glucose PD solutions during heat sterilization [12]. In fact, when peritoneal membrane cells are exposed to these solutions, high glucose and GDP concentrations are further chemically modified to become AGEs, whose accumulation in peritoneal cells induces peritoneal cell oxidative and inflammatory injury [12].

The glucose and lactate present in conventional PD solutions and the accumulation in the dialysate of glucose degradation products (GDPs), in fact, induces AGEs, other pro-oxidants and inflammatory substances in peritoneal cells, increasing, as mentioned above, OxSt in PD patients. Increased OxSt might, in these patients, induce medium–long-term structural and functional damage of the peritoneal membrane. These changes include progressive submesothelial thickening, the narrowing and hyalinization of the vascular lumen, the thickening of the basal capillary membrane and of the arterial wall, increased synthesis of proinflammatory cytokines and ROS, the inhibition of cell growth and proliferation and DNA damage [14,15]. In addition, the long-term exposure of peritoneal mesothelial cells to the high glucose concentrations of PD solutions can cause morphological and functional alterations similar to those that occur during the epithelial to mesenchymal transition [16,17,18]. Furthermore, the high glucose concentrations in PD solutions have been shown to result in an increased intraperitoneal production of IL-6, which has a fibrogenic activity via JAK/STAT3 signaling and TGF-β/Smad-3 pathways [18].

The peritoneal membrane damage usually manifests with a progressive reduction in ultrafiltration volume and later with the loss of depuration ability causing the patient to drop out of this RRT technique to switch to hemodialysis.

In fact, in a recent study using a molecular biology approach, we found that OxSt in PD patients was significantly increased [19]. This was proven in terms of increased levels of p22^phox^, a subunit of NADPH oxidase that is essential to form O_2_^•−^ [20]; increased ROCK activity in terms of increased myosin-phosphatase target protein-1 (MYPT-1) phosphorylation, which is deeply involved in OxSt generation [21] and increased ferritin as a marker of inflammation after 6 months of PD [19].

## 3. Icodextrin and the Need for More Biocompatible PD Solutions

All the above-mentioned findings call to attention the need for more biocompatible dialysis solutions with different glucose polymers to prevent and/or treat OxSt and inflammation.

More biocompatible dialysis solutions have been developed [22,23] that have a pH closer to physiologic pH, and bicarbonate as buffer, which induces low amounts of GDPs and AGEs. However, the high osmolarity of solutions remains a crucial problem that contributes to the increase in OxSt.

The most known and used of such glucose-free and more biocompatible PD solutions is icodextrin. Icodextrin is a water-soluble polymer, which induces a slow but sustained rate of peritoneal ultrafiltration. The benefits of icodextrin include fewer episodes of fluid overload and improved peritoneal ultrafiltration [24]. In addition, due to its higher biocompatibility, icodextrin can protect the integrity of the peritoneal membrane via a reduction in OxSt [25] and is associated with a better control of lipids, glucose and blood pressure. However, although its higher biocompatibility compared to glucose-based PD solutions could predict a favorable effect on OxSt, earlier studies in PD showed conflicting evidence regarding the impact of icodextrin-based solutions on chronic inflammation and OxSt [12]. Although, in fact, icodextrin-based solutions were shown to induce a reduction in AGEs and of various markers of carbonyl oxidation in vitro, the exposure of mice peritoneal membrane cells to icodextrin resulted instead in an accelerated lipid peroxidation status detected in the peritoneal-drained effluent [12]. A significant protective effect on OxSt through the use of icodextrin-based glucose-free solutions in PD patients was instead clearly demonstrated by a very recent study, which used a molecular biology approach [26]. In this study, the reduction in OxSt was in fact documented by the significant reduction in ROCK activity, in terms of monocyte MYPT-1 phosphorylation, with a 9% statistically significant reduction after 3 months from the start of the dialysis procedure using icodextrin solution compared to the baseline, and a further statistically significant decrease up to 15% after 6 months [26]. OxSt reduction in PD patients under icodextrin treatment was also documented by a significant reduction in lipid peroxidation in terms of the production of MDA, a reactive aldehyde produced in the presence of O_2_^•−^, both at 3 months compared to baseline (−13%) and after 6 months compared to both baseline and 3 months (−7%) of icodextrin treatment [26]. It should be underlined that this study was carried out on a very small cohort of patients (15 patients for the first 3 months and 9 patients continued until the 6 month timepoint) [26]; however, this study may be considered as a working hypothesis for a larger cohort and with a longer duration. In fact, if was confirmed by larger studies that this evidence would represent a starting point for the change in our current practice in peritoneal dialysis prescription and promote a higher utilization of glucose-free-based solutions to prevent the oxidative stress damage of the peritoneal membrane, thereby prolonging the use of this RRT technique.

In clinical practice, only two osmotic agents are currently available in glucose-free solutions for PD—icodextrin and amino acids. The improvement in the biocompatibility of PD solutions might, therefore, represent a promising strategy to reduce/suppress OxSt and OxSt-related inflammatory/fibrogenic activity, preserving the integrity of peritoneal membrane and improving patients’ outcomes [14]. The study of novel tools to fight glucose-associated abnormalities such as the use of osmo-metabolic agents in the PD solutions is heading in this direction [15].

## 4. Osmo-Metabolites: The Promising Solution

Osmo-metabolites are substances that have favorable osmotic and metabolic properties. This approach—the use of bioactive glucose-sparing agents—would allow glucose-free or glucose-sparing dialysis fluids not only to reduce glucose exposure in the peritoneum without compromising ultrafiltration, but also to independently reduce the underlying systemic negative metabolic effects caused by the glucose load. Two such candidate agents (L-carnitine and xylitol) in PD fluids are a new glucose-sparing strategy, and studies with these substances used alone, or in combination, have shown promising results [27]. In fact, these PD solutions have shown preliminary competitive potential regarding efficiency, efficacy and safety [27].

XyloCore, an association of L-carnitine, Xylitol and low glucose (27.7 mmol/L), seems to have the same ultrafiltration ability of the high concentration of glucose without the deleterious effect of the latter [14,15]. In fact, contrary to solutions at higher glucose concentrations, treatment with XyloCore maintained the viability of mesothelial and endothelial cells 153] and does not exert profibrotic, inflammatory and angiogenic effects [15].

The results of the ongoing FIRST and ELIXIR studies will likely provide evidence on the positive approach to PD treatment with osmo-metabolic agents.

The FIRST trial (NCT04001036) evaluates the efficacy and safety assessments of a PD solution containing glucose, Xylitol and L-Carnitine compared to standard PD solutions in CAPD; the ELIXIR trial evaluates the efficacy and safety of Xylocore, in an international multicenter 6 month study (NCT03994471). These studies will examine the safety, tolerability and efficacy of the new PD solutions based on L-carnitine, Xylitol and low-glucose not only on the preservation of the peritoneal membrane and residual kidney function, but also on underlying cardiovascular comorbidities, which increase cardiovascular risk (Table 1).

Finally, future research should also be performed to identify additional osmo-metabolic agents and how to combine them for the better preservation of the peritoneal membrane, residual kidney function and underlying risk factors for cardiovascular disease.

## 5. Conclusions

OxSt in PD patients is significantly further increased compared to the already activated OxSt of their pre-dialysis phase. In particular, this is due to the use of glucose or lactate-based fluids that are currently used in peritoneal dialysis, which may lead to important intra-peritoneal and systemic clinical consequences. There is, therefore, the need for more biocompatible peritoneal dialysis solutions to reduce/abolish the oxidative stress induced by glucose-based peritoneal dialysis solutions and its damaging effects on the peritoneal membrane. The reduction in oxidative stress shown by icodextrin-based peritoneal dialysis solutions supports the possibility in clinical practice of a more widespread use of icodextrin-based fluids. In addition, the reduction in oxidative stress obtained in peritoneal dialysis with icodextrin-based solutions points towards the replacement of the glucose-based fluids currently used in order to better preserve peritoneal membrane integrity and residual function via protection from oxidative stress and oxidative stress-mediated inflammation/fibrosis. This paradigm also supports the rationale of the ongoing studies using osmo-metabolic agent-based fluids in peritoneal dialysis such as those based on L-carnitine, xylitol and their combination. The results of these studies could soon provide valuable information for a further step forward not only to preserve peritoneal membrane integrity and residual renal function, but also to reduce CV disease risk factors such as oxidative stress itself and those mediated by oxidative stress such as hypertension and CV disease.

## Figures and Tables

**Table 1:** Studies reporting beneficial effects of peritoneal dialysis glucose-free solutions.

Study	Year	Glucose-Free Solution	Sample Size	Oxidative Stress Biomarkers	Results
Basso A et al.	2024 [26]	Icodextrin	in vivo, 15 peritoneal dialysis incident patients (3 months); 8 patients (6 months)	Circulating p22^phox^ MYPT-1 phosphorylation state MDA, IL-6	p22phox, MYPT 1 phosphorylation and MDA reduced after 3 months of icodextrin; MYPT-1 and MDA further reduced after 6 months. IL-6 has no statistically significant reduction.
Bonomini M et al.	2016 [28]	Xylitol–Carnitine–low glucose vs. glucose	in vitro (human endothelial cellls)	Peroxynitrite Cell viability VCAM-1, ICAM-1	Xylitol–Carnitine–low glucose: improved endothelial cell viability; glucose solutions increased intra-cellular peroxynitrite levels, VCAM-1 and ICAM-1 exposure and interactions with monocytes compared to the experimental solution.
Yung S et al.	2015 [29]	Low glucose peritoneal dialysis regimen (PEN = Physioneal, Extraneal, Nutrineal) vs. glucose-based dialysis solutions (control)	in vivo, 150 incident peritoneal dialysis patients	Serum and dialysate decorin, HGF, VEGF, HA, ICAM, VCAM-1, P-selectina, IL-6, TNF-alfa, CA125	Dialysate CA125: higher in PEN, decreased after switch to control. Serum decorin, HGF, VEGF: higher in PEN group after 12 months, but after switch to glucose-based solutions, no difference. Dialysate IL-6: higher in PEN after 12 months, even more after switch to glucose-based solutions. Serum adiponectine: higher in PEN after 12 months; decreased slightly after switch to glucose-based solutions. Dialysate s-ICAM and VCAM-1: higher in PEN after 12 months and decreased after switch to glucose-based solutions. VEGF: no difference. Urine volume: higher in PEN regimen; creatinince clearance: higher in PEN regimen < D/p creatinine: increased in PD. Dialysate decorin, IL-6, HA, s-ICAM, P-selectin: higher in dialysate compared to serum.
Bonomini M et al.	2011 [30]	L-Carnitine	in vitro (HUVECs and murine fibroblasts)	AQP1 expression water transport and cell viability glucose-induced apoptosis	Exposure to high glucose medium resulted in decrease in AQP1 levels; the addition of L-carnitine to D-glucose reverted the inhibitory effect of glucose on AQP1 expression; the addition of L-carnitine to either 1.5 or 2.5% glucose improved fibroblasts’ viability.
Masola V et al.	2010 [15]	Xylitol (low and medium strength) vs. 1.36% and 2.27% glucose-based solutions	in vitro (HUVECs, human peritoneal mesothelial cells (HMRSV5), human microvascular endothelial cell line)	Mesothelial–mesenchimal transition, TGF-beta and SNAI1 gene expression; IL-6, IL-1beta, TNF-alfa, VEGF gene and protein expressions	Reduction in viability of HUVEC and HMRSV5 cells after exposure to 2.27% glucose-based solutions; morphology change in mesothelial and endothelial cells after exposure to glucose-based solutions; near normal morphology with xylitol-based solutions; TGF-beta and SNAI1 expression increased with glucose-based solutions, not with low-strength xylitol solutions and mildly with medium-strength xylitol solutions; increase in alfa-SMA and VIM gene expression with glucose-based solutions; xylitol solutions did not affect IL-6, IL-1 beta gene and protein expression; xylitol medium strength increased VEGF gene expression on endothelial cells and release in mesothelial cells but lower than glucose-based solutions.
Mortier S et al.	2004 [31]	Amino acid vs. glucose-based vs. low glucose-based solutions	in vivo 48 female rats	AGEs, RAGE, VEGF on peritoneum	eNOS and VEGFF, AGE expression and submesothelial fibrosis were higher in standard glucose-based solution group.
Ueda Y et al.	2000 [25]	Icodextrin or amino acid vs. glucose-based solutions	in vivo, 6 patients already on peritoneal dialysis from an average of 26 months	Effluent AGEs, reactive carbonyl compounds	Reactive carbonyl compounds lower in icodextrin and amino acid group, but difference decreases progressively during dwell time.

## Data Availability

No new data were created or analyzed in this study. Data sharing is not applicable to this article.
